# Early cardiovascular collapse after envenoming by snakes in Australia, 2005–2020: an observational study (ASP‐31)

**DOI:** 10.5694/mja2.52622

**Published:** 2025-03-09

**Authors:** Geoffrey K Isbister, Katherine Z Isoardi, Angela L Chiew, Shane Jenkins, Nicholas A Buckley

**Affiliations:** ^1^ The University of Sydney Sydney NSW; ^2^ The University of Newcastle Newcastle NSW; ^3^ NSW Poisons Information Centre Children's Hospital at Westmead Sydney NSW; ^4^ Princess Alexandra Hospital Brisbane QLD; ^5^ The University of Queensland Brisbane QLD; ^6^ Prince of Wales Hospital and Community Health Services Sydney NSW

**Keywords:** Snake bites, Hypotension, Coagulation disorders

## Abstract

**Objectives:**

To investigate the frequency, timing, and characteristics of cardiovascular collapse after snakebite in Australia, and the complications of collapse following envenoming.

**Study design:**

Observational study; analysis of prospectively collected demographic and clinical data.

**Setting, participants:**

People with confirmed snake envenoming recruited to the Australian Snakebite Project at one of 200 participating Australian hospitals, 1 July 2005 – 30 June 2020.

**Main outcome measures:**

Time from snakebite to collapse; post‐collapse complications (cardiac arrest, seizures, death).

**Results:**

Of 1259 envenomed people, 157 (12%) collapsed within 24 hours of the snakebite; venom‐induced consumption coagulopathy (VICC) was determined in all 156 people for whom coagulation testing could be performed. The exact time between bite and collapse was known for 149 people (median, 20 min; interquartile range, 15–30 min; range, 5–115 min); the time exceeded 60 minutes for only two people, each after releasing tight bandages 60 minutes after the bite. The collapse preceded hospital arrival in 132 cases (84%). Brown snake (*Pseudonaja* spp.) envenoming was the leading cause of collapse (103 cases, 66%). Forty‐two collapses (27%) were followed by cardiac arrest, 49 (31%) by seizures (33 without cardiac arrest), and five by apnoea; collapse was associated with hypotension in all 24 people whose blood pressure could be measured at or close to the time of collapse. Twenty‐five people who collapsed died (16%), and seven of the envenomed people who did not collapse (0.6%; difference: 15 percentage points; 95% confidence interval, 8–21 percentage points). The deaths of 21 of the 25 people who collapsed were immediately associated with the cardiac arrest that followed the collapse; three people who did not have cardiac arrests died later of intracranial haemorrhage, and one of hyperthermia. The proportion of people who had collapsed before reaching hospital was larger for people who died of post‐collapse cardiac arrest (13 of 21, 62%) than for those who survived (6 of 21, 28%).

**Conclusion:**

Collapse after Australian snake envenoming almost always occurred within 60 minutes of the bite, was always accompanied by VICC, and most frequently followed brown snake bites. Poorer outcomes, including cardiac arrest, seizures, and death, were more frequent for people who collapsed than for those who did not. Outcomes for people who collapsed before medical care arrived were poorer than for those who collapsed in hospital or in an ambulance.

**The known**: Collapse is an infrequent but important manifestation of snake envenoming in Australia.

**The new**: People who collapse after snake envenoming in Australia do so within an hour of the bite and usually before they reach hospital. It is associated with venom‐induced consumption coagulopathy, and is most frequent after brown snake bites. Rates of cardiac arrest and death are higher for people who collapse than for those who do not.

**The implications**: Early collapse is a high risk feature of Australian snake envenoming. It requires prompt identification and cardiopulmonary resuscitation.

Snakebite causes substantial numbers of injuries and deaths around the world,^1^, ^2^ including in Australia.^3^ Australian snake envenoming causes a constellation of clinical effects that depend on the snake type and the venom injected. Venom‐induced consumption coagulopathy (VICC) is the most frequent effect, followed by thrombotic microangiopathy, myotoxicity, and neurotoxicity.^3^, ^4^ An unusual effect of envenoming by Australian elapids is collapse within an hour of the bite or loss of consciousness;^4^ this is the most frequent mechanism of death following snakebite in Australia.^3^

Early collapse, cardiovascular collapse, or hypotensive collapse have been reported following bites by brown snakes,^5^ tiger snakes (*Notechis scutatus*),^6^ rough‐scale snakes (*Tropidechis carinatus*),^7^ and taipans (*Oxyuranus scutellatus*).^8^ Collapse or, more frequently, hypotension have been reported following snakebite in other parts of the world, mainly after viper envenoming,^9^, ^10^ but from a variety of different mechanisms.^11^, ^12^

The cause of collapse following bites by Australian elapids has been debated. It has been attributed to a myocardial depressant in the venom or intracardiac clot formation,^13^, ^14^ but more recent studies in rodents suggest that the mechanism involves the secondary release of vasodilatory substances.^15^, ^16^ Whatever the mechanism, hypotensive collapse occurs within minutes in experimental animals, and within one hour of envenoming in humans.^3^

A recent New South Wales coronial report focused attention on early collapse and death following brown snake envenoming.^17^ The coroner recommended several changes to the observation of patients, as well as considering administering antivenom to people not exhibiting signs of envenoming. However, the published information about the timing of the collapse, the risk of cardiac arrest, seizure, and death in this case was limited. These factors are critical for the probable effectiveness of antivenom in this situation.

We therefore investigated the frequency, timing, and characteristics of cardiovascular collapse after snakebite in Australia, and the complications of collapse following envenoming.

## Methods

To assess the characteristics of and complications in people who experienced cardiovascular collapse following snakebite and to compare them with those for other people who had been envenomed, we reviewed cases in the Australian Snakebite Project (ASP) cohort. The Australian Snakebite Project (ASP) is a prospective, multicentre observational study that recruits people with suspected snakebite.

All people who presented to any of more than 200 participating Australian hospitals with suspected or confirmed snakebite during 1 July 2005 – 30 June 2020 were eligible for recruitment by ASP investigators. People with suspected or confirmed snakebite are identified by calls to a national free call number, calls to the National Poison Centre Network, by clinical toxicologists, or hospital investigators. When a case is identified, consent, patient information, and datasheet forms are faxed or emailed to the treating doctor. Demographic data and information about the circumstances of the bite, clinical effects, laboratory investigations, complications, and treatment are collected.^3^ The information is entered into Microsoft Access, and any missing information is obtained from the patient's medical record. All cases are reviewed by the chief investigator (author GKI). An additional blood sample is collected, and the serum frozen and transported for venom assays when available. The snake type is determined by expert identification (professional working with snakes at a museum or zoo) when the snake is available, or by venom‐specific enzyme immunoassay when blood is available.^18^

For each case, we extracted patient demographic data and information on bite circumstances, the timing of collapse (if applicable), time to hospital arrival, clinical syndromes of Australian snake envenoming (VICC, myotoxicity, neurotoxicity),^3^ complications (cardiac arrest, seizure, death, thrombotic microangiopathy), length of stay, and antivenom treatment. Collapse was defined as any physical collapse in an upright person associated with loss of consciousness (ie, not simply a fall) or any loss of consciousness in a recumbent patient (eg, on an ambulance stretcher or hospital bed) within 24 hours of snakebite. We excluded people for whom there was clear evidence of collapse associated with venom hypersensitivity or anaphylaxis, a rare event confined to snake handlers.^19^

We summarise continuous variables as medians with interquartile ranges (IQRs) and ranges. The statistical significance of differences in dichotomous outcomes was assessed in Fisher exact tests, that of differences in continuous variables with Mann–Whitney tests. All analyses were performed in GraphPad Prism 9.5 for Windows.

### Ethics approval

The study was approved by human research ethics committees of the Northern Territory Department of Health and Menzies School of Health Research (04/08), the Hunter New England Local Health District (HREC/15/HNE/29), the Royal Perth Hospital and South Metro Area Health Service (RA‐08/003), the Western Australian Country Health Service (2008: 03, REC200835), Tasmania Network (H00109965), and the Gold Coast Health Service District. (200835).

## Results

A total of 2180 people with snakebite were recruited to ASP during 2005–20; 1259 had been envenomed, of whom 157 (12%) subsequently collapsed (Box 1). Of the 157 people who experienced collapse, 125 (80%) were male, their median age was 43 years (IQR, 26–56 years), and 20 (13%) were snake handlers; the characteristics of envenomed people who did not collapse were similar (Box 2). The proportions of envenoming cases followed by collapse were similar by state and territory, remoteness category of hospital (Box 2) and by year (Supporting Information, figure 1).

Box 1 **Flow chart of people recruited for the Australian Snakebite Project, 1 July 2005 – 30 June 2020, by envenoming, post‐envenoming collapse, and post‐collapse cardiac arrest status**

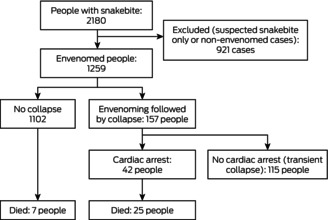

Box 2 **Characteristics of the 1259 envenomed people recruited for the Australian Snakebite Project, 1 July 2005 – 30 June 2020, and their snakebites, by whether they collapsed within 24 hours of the snakebite**

**Table:** 

Characteristic	Collapse	No collapse
Number of people	157	1102
Age (years), median (IQR)	43 (26–56)	41 (24–57)
Sex		
Male	125 (80%)	793 (72%)
Female	32 (20%)	308 (28%)
Snake handler	20 (13%)	182 (17%)
State/territory		
Australian Capital Territory	1 (1%)	10 (1%)
New South Wales	47 (30%)	393 (36%)
Northern Territory	9 (6%)	34 (3%)
Queensland	60 (38%)	317 (29%)
South Australia	3 (2%)	18 (2%)
Tasmania	4 (3%)	27 (2%)
Victoria	13 (8%)	141 (13%)
Western Australia	20 (13%)	162 (15%)
Geographic remoteness (hospital)^20^		
Major city	43 (27%)	409 (37%)
Inner regional	63 (40%)	447 (41%)
Outer regional	24 (15%)	152 (13%)
Remote	21 (13%)	59 (5%)
Very remote	5 (3%)	35 (3%)
Snake type		
Brown snake (*Pseudonaja*)	103 (66%)	280 (25%)
Tiger snake (*Notechis*)	12 (8%)	179 (16%)
Rough‐scale snake (*Tropidechis carinatus*)	12 (8%)	78 (7%)
Tiger snake group*	5 (3%)	44 (4%)
Taipan (*Oxyuranus scutellatus*)	4 (3%)	51 (5%)
Broad‐headed snake (*Hoplocephalus*)	2 (1%)	34 (3%)
Black snake (*Pseudechis*)	0	240 (22%)
Death adder (*Acanthophis*)	0	37 (3%)
Other	0	20 (2%)
Unknown	19 (12%)	139 (13%)
Clinical syndromes		
Venom‐induced consumption coagulopathy	156 (99%)	757 (69%)
Thrombotic microangiopathy	8 (5%)	106 (10%)
Neurotoxicity	4 (3%)	119 (11%)
Myotoxicity	7 (4%)	190 (17%)
Hospital length of stay (hours), median (IQR)	43 (26–75)	38 (22–65)
Antivenom administered	150 (96%)	903 (82%)

IQR = interquartile range. *Tiger snake or rough‐scaled snake (unable to distinguish with venom‐specific enzyme immunoassay).

### Characteristics of cases of envenoming followed by collapse

One hundred and three of the 157 people who collapsed (66%) were envenomed by brown snakes, 29 (19%) by tiger snakes (*Notechis* spp.) or rough‐scaled snakes (*T. carinatus*), four (5%) by taipans (*Oxyuranus* spp.), and none by death adders (*Acanthophis* spp.) or black snakes (*Pseudechis* spp.); the snake was not identified in nineteen cases (12%). In cases not followed by collapse, the proportion of envenomings by brown snakes was smaller (280 cases, 25%), and 240 cases involved black snakes (22%) (Box 2). Collapse was more frequent following brown snake envenoming (103 of 383 cases, 27%) than envenoming by tiger or rough‐scale snakes (29 of 330, 9%) or taipans (4 of 55, 7%). VICC was determined in all 156 patients for whom coagulation tests were performed (one person who collapsed died before reaching a hospital). Four people experienced neurotoxicity, and seven myotoxicity. There was evidence of venom hypersensitivity in one patient who collapsed, and they were excluded from further analyses.

The median time from snakebite to collapse in the 149 cases for which time of collapse was known was 20 minutes (IQR, 15–30 min; range, 5–115 min) (Box 3). The two people who collapsed more than 60 minutes after the bite (90 or 115 minutes) did not have any suggestive symptoms, and coagulation study findings were normal on arrival at hospital (respectively 70 and 30 minutes after the bite), but they collapsed after their bandage was removed; one was a snake‐handler who had applied a double pressure bandage immediately after the bite. Collapse preceded arrival at the hospital in 132 cases (84%). A pressure bandage with immobilisation had been applied to 130 of 157 people (83%) who collapsed; it had been applied before the collapse in 50 cases (32%) at a median of ten minutes (IQR, 5–15 min) after the bite. No patient who collapsed had received antivenom prior to collapse.

Box 3 Distribution of time to hospital and to collapse, by site of collapse*

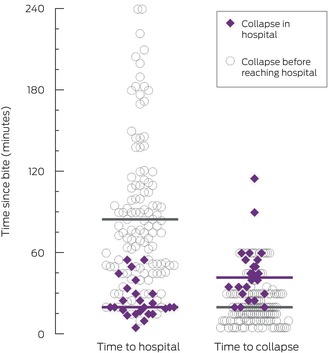

* Purple bars mark the median values for 25 people who collapsed in hospital, grey bars for the 132 people who collapsed before reaching hospital.

### Complications of collapse

Forty‐two collapses (27%) were followed by cardiac arrests, 49 (31%) by seizures (33 without cardiac arrest), and five by apnoea (Box 4). Two patients were intubated following collapse because of agitation, one because of hypotension. Collapse was associated with hypotension in all 24 people whose blood pressure could be measured at or close to the time of collapse. All people who collapsed without complications before arriving at hospital arrived with recovered or recovering level of consciousness. Twenty‐one children (under 18 years) collapsed; eleven (52%) had seizures, eight had cardiac arrests (38%) (Box 4).

Box 4 **Complications, deaths, and intubation for 157 people who collapsed after snakebite envenoming, by age group**

**Table:** 

Outcome	Collapse	Adults	Children
Number of patients	157	136	21
Seizure	49 (31%)	38 (28%)	11 (52%)
Cardiac arrest	42 (27%)	34 (25%)	8 (38%)
Respiratory arrest (apnoea)	5 (3%)	5 (4%)	0
Died	25 (16%)	22 (16%)	3 (14%)
Intubated (immediately after collapse)	37 (24%)	32 (24%)	5 (24%)

Twenty‐five people who collapsed (16%) died, as did seven (0.6%) who did not collapse (difference: 15 percentage points; 95% confidence interval, 8–21 percentage points). The deaths of 21 of the 25 people who collapsed were immediately associated with the cardiac arrest that followed the collapse; four people who did not have cardiac arrests died later from intracranial haemorrhage (three people) or hyperthermia (one person). Among the 42 people who collapsed and had cardiac arrests, return of spontaneous circulation was reported in thirty; median time to return of spontaneous circulation was shorter for the eighteen who survived (5 min; IQR, 2–6 min; range, 1–40 min) than for the twelve who died (45 min; IQR, 32–60 min; range, 15–120 min; *P* < 0.001). Thirteen of the 21 people who died following cardiac arrests had collapsed before reaching hospital, six in the ambulance or in hospital; twelve of the 21 who survived had collapsed in the ambulance or in hospital (Supporting Information, table 1).

## Discussion

We found that collapse following snakebite usually occurs soon after the bite and is associated with more severe outcomes, including cardiac arrest and death, than envenoming not followed by collapse. Collapse was most frequent after brown snake envenoming, and was always accompanied by VICC. As the median time to collapse was 20 minutes, and it was within 60 minutes of the bite in 99% of cases, 84% of collapses took place before the person arrived at hospital. Antivenom had never been administered before the collapse, indicating the importance of immediate resuscitation after severe snake envenoming. This finding was reinforced by the fact that 19 of the 21 people who collapsed and survived cardiac arrests had received immediate resuscitation because they were in hospital or with ambulance officers, or had received immediate bystander resuscitation (Supporting Information, table 1).

The recent coronial report on a death following collapse in hospital after brown snake envenoming recommended that antivenom be administered as soon as evidence of envenoming is noted, based on medical assessment, even if laboratory results are not yet available.^17^ However, we found that 84% of collapses preceded arrival at hospital, and in no case could antivenom be administered before a collapse. As collapse typically occurs early, and is often the first indication of envenoming, administering antivenom to prevent collapse is practically very difficult. More importantly, we found that recognising collapse and immediate resuscitation are crucial, and poor outcomes were more frequent when resuscitation, and consequently return of spontaneous circulation, were delayed.

More than one‐quarter of brown snake envenomings were followed by collapse, a larger proportion than for tiger snake or taipan envenomings. Envenoming by snakes that do not cause VICC (death adders and black snakes) was not followed by collapse. That is, collapse is associated with more severe envenoming, but it also suggests brown snake envenoming, particularly in the absence of neurotoxicity or myotoxicity.

### Limitations

Limitations of the study included uncertainty about the time of collapse, difficulties in measuring blood pressure or other vital characteristics at the time of the collapse, and some collapses not being witnessed. Time of collapse was based on the report to the admitting doctor, ambulance or other health care workers. Further, people who presented to hospital after a delay might not have recalled a collapse, particularly if it was not witnessed. However, the fact that collapse occurred early meant that most people who collapsed were transported to hospital immediately. As no‐one received it early enough, the effectiveness of antivenom for preventing collapse is unknown; its administration prior to reaching hospital seems, in any case, to be impractical. We could not assess the effect of other medical conditions on collapse because this information is not routinely collected in ASP. Finally, people who collapsed may have been more likely to enrol in ASP than people who did not.

### Conclusion

Collapse after Australian snake envenoming almost always occurred within 60 minutes of the bite, and was always accompanied by VICC; it most frequently followed brown snake bites. Collapse is associated with poorer outcomes than for people who do not collapse after snakebite, including higher rates of cardiac arrest and death, particularly if resuscitation is delayed. A larger proportion of people who survived subsequent cardiac arrests than of those who did not had collapsed in the ambulance on the way to hospital or in hospital, and their median time to return of spontaneous circulation was shorter. We propose that “early collapse” be used to describe this common and potentially lethal phenomenon: collapse within one hour of envenoming, or within two hours if a pressure bandage has been applied. This definition could be useful for assessing its frequency after snakebite elsewhere in the world.

### Open access

Open access publishing facilitated by The University of Newcastle, as part of the Wiley – the University of Newcastle agreement via the Council of Australian University Librarians.

### Competing interests

No relevant disclosures.

## Supporting information


Supplementary results
