# Supplementary material for: Early cardiovascular collapse after envenoming by snakes in Australia, 2005–2020: an observational study (ASP‐31)
Source: Med J Aust. 2025 Mar 9;222(6):313–7. doi: 10.5694/mja2.52622 (PMC11972595; doi:10.5694/mja2.52622)
Supplement: Supplementary file 1 — Supplementary results [file MJA2-222-313-s001.pdf]

The Medical Journal of Australia

Supporting Information

Supplementary results

This appendix was part of the submitted manuscript and has been peer reviewed.
It is posted as supplied by the authors.

Appendix to: Isbister GK, Isoardi KZ, Chiew AL, et al. Early cardiovascular collapse after envenoming by snakes in Australia, 2005–2020: an observational study (ASP-31). *Med J Aust* 2025; doi: 10.5694/mja2.52622.

Figure 1. Numbers of envenomed people recruited for the Australian Snakebite Project, 1 January 2006 – 31 December 2019, and of cases followed by collapse cases, by year*

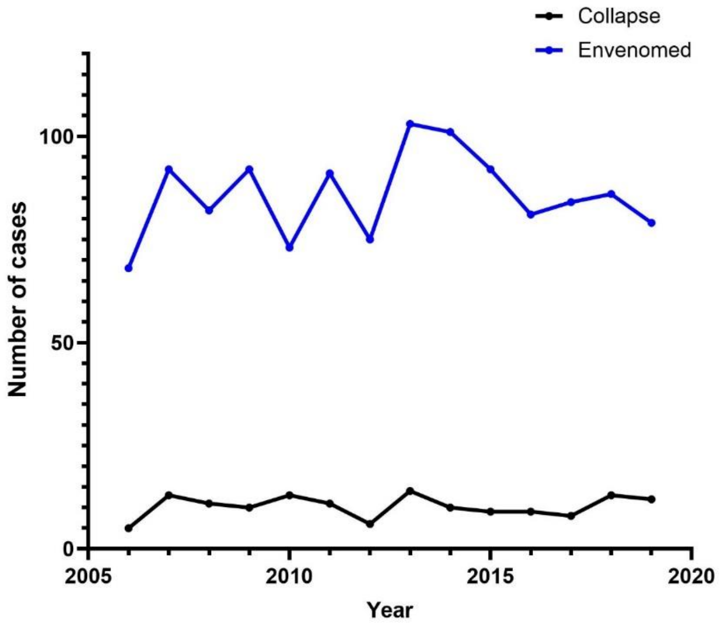

* Numbers for 2005 and 2021 are omitted because data were available for only six months in each case.

Table 1. Location of collapse for 42 people who subsequently had cardiac arrests, by whether they survived

Site of collapse	Survived	Died
Number of people	21	21
Ambulance	4 (19%)	3 (14%)
Hospital	8 (38%)	3 (14%)
Out-of-hospital (bystander CPR)	7 (33%)	2 (10%)
Out-of-hospital (no attempt at CPR)	2 (10%)	13 (62%)

CPR = cardiopulmonary resuscitation. Bystander CPR: the person received almost immediate and apparently effective CPR from a bystander.

STROBE Statement—checklist of items that should be included in reports of observational studies. Note: The page numbers refer to the submitted manuscript, not to the published article or its supplementary file.

	Item No.	Recommendation	Page No.	Relevant text from manuscript
**Title and abstract**	1	(*a*) Indicate the study's design with a commonly used term in the title or the abstract	1	Prospective, multicentre cohort study of …
		(*b*) Provide in the abstract an informative and balanced summary of what was done and what was found	1	We found that collapse occurred early and was associated with VICC, the majority brown snake bites. Collapse associated with poor outcomes, including cardiac arrest, seizures and death. Improved outcomes were associated with collapses occurring in hospital.
**Introduction**				
Background/rationale	2	Explain the scientific background and rationale for the investigation being reported	3	An unusual effect from Australian elapid envenoming is collapse within an hour of the bite, or loss of consciousness.[4] This is the commonest mechanism of death in Australian snakebite.[3]<br>In Australia, collapse has been reported in bites from brown snakes, tiger snake (Notechis scutatus), rough-scale snake (Tropidechis carinatus) and taipan (Oxyuranus scutellatus).[5-8] It has variously been described as early collapse, cardiovascular collapse or hypotensive collapse. Collapse, or more often hypotension, has been reported in snakebites from other parts of the world, mainly in viper envenoming, [9,10] although these effects have a range of different mechanisms.[11, 12]
Objectives	3	State specific objectives, including any prespecified hypotheses	3	We aimed to describe the frequency, timing and characteristics of patients with collapse, and complications of collapse in Australian snake envenoming.
**Methods**				
Study design	4	Present key elements of study design early in the paper	4,5	ASP is a prospective cohort study of all snakebite cases recruited from over 200 hospitals across Australia. The design of ASP has been previously reported.[3]
Setting	5	Describe the setting, locations, and relevant dates, including periods of recruitment, exposure, follow-up, and data collection	5	For this study we included all envenomed patients with and without a reported collapse following a snakebite from July 2005 to June 2020.
Participants	6	(*a*) *Cohort study*—Give the eligibility criteria, and the sources and methods of selection of participants. Describe methods of follow-up<br>*Case-control study*—Give the eligibility criteria, and the sources and methods of case ascertainment and	5	Patients with a suspected or confirmed snakebite are identified by calls to a national free call number, calls to the National Poison Centre Network, by clinical toxicologists or hospital investigators. We excluded patients for which there was clear evidence of a collapse associated with hypersensitivity/anaphylaxis, a rare event confined to snake handlers.[19]

		control selection. Give the rationale for the choice of cases and controls<br>*Cross-sectional study*—Give the eligibility criteria, and the sources and methods of selection of participants		
		(*b*) *Cohort study*—For matched studies, give matching criteria and number of exposed and unexposed<br>*Case-control study*—For matched studies, give matching criteria and the number of controls per case		N/A
Variables	7	Clearly define all outcomes, exposures, predictors, potential confounders, and effect modifiers. Give diagnostic criteria, if applicable	5	Collapse was defined as any physical collapse in a person upright, associated with a loss of consciousness (i.e. not simply a fall), or any loss of consciousness in a recumbent patient (e.g. on an ambulance stretcher or hospital bed), within 24 hours of snakebite.
Data sources/ measurement	8*	For each variable of interest, give sources of data and details of methods of assessment (measurement). Describe comparability of assessment methods if there is more than one group	5	In each case we extracted patient demographics, bite circumstances, presence and timing of collapse, time to hospital arrival, clinical syndromes of Australian snake envenoming (VICC, myotoxicity and neurotoxicity)[3], complications (cardiac arrest, seizure, death, thrombotic microangiopathy), length of stay (LOS) and antivenom treatment.
Bias	9	Describe any efforts to address potential sources of bias	5	
Study size	10	Explain how the study size was arrived at	5	Based on the cohort size recruited.
Quantitative variables	11	Explain how quantitative variables were handled in the analyses. If applicable, describe which groupings were chosen and why	5	Medians, interquartile ranges (IQR) and ranges are reported for all continuous variables. Dichotomous variables are reported with 95% confidence intervals (CI), estimated using Wilson's procedure and continuity correction. Differences in dichotomous outcomes were compared using Fisher's exact test. Continuous variables were compared using Mann–Whitney test. All graphs and analyses were done using GraphPad Prism version 9.5 for Windows (GraphPad Software, San Diego California USA, www.graphpad.com).
Statistical methods	12	(*a*) Describe all statistical methods, including those used to control for confounding	5	
		(*b*) Describe any methods used to examine subgroups and interactions	5	
		(*c*) Explain how missing data were addressed	N/A	

		(*d*) *Cohort study*—If applicable, explain how loss to follow-up was addressed<br>*Case-control study*—If applicable, explain how matching of cases and controls was addressed<br>*Cross-sectional study*—If applicable, describe analytical methods taking account of sampling strategy	N/A	
		(*e*) Describe any sensitivity analyses	N/A	
**Results**				
Participants	13*	(a) Report numbers of individuals at each stage of study—eg numbers potentially eligible, examined for eligibility, confirmed eligible, included in the study, completing follow-up, and analysed	5, Fig 1	There were 2180 cases recruited to ASP from July 2005 to June 2020 and 1259 snakebite patients were envenomed (Figure 1). There were 157 (12%) envenomed patients that had a collapse and 1102 envenomed patients who did not have a collapse.
		(b) Give reasons for non-participation at each stage	N/A	
		(c) Consider use of a flow diagram	Fig 1	
Descriptive data	14*	(a) Give characteristics of study participants (eg demographic, clinical, social) and information on exposures and potential confounders	5	Of the 157 with collapse, 125 (80%) were male, and the median age was 43 years (interquartile range [IQR]: 26 to 56 years) and 20 (13%) were snake handlers, which was similar to envenomed patients without collapse (Table 1). The number of collapse was similar for different States/Territories, different locations of the hospital (city, regional centre or remote location) and over the duration of the study (Supplementary Table 1; Supplementary Figure 1).
		(b) Indicate number of participants with missing data for each variable of interest	N/A	
		(c) *Cohort study*—Summarise follow-up time (eg, average and total amount)	4,5,6	Treatment in hospital with envenoming
Outcome data	15*	*Cohort study*—Report numbers of outcome events or summary measures over time	*5*	*In this cohort of 157 patients with collapse, 42 (27%) had a subsequent cardiac arrest, 49 (31%) had a seizure (33 without a cardiac arrest) and five had apnoea (Table 2). A further three patients were intubated following collapse for agitation (2) and hypotension (1). The collapse was associated with hypotension in all 24 patients in which a blood pressure could be measured at or close to the time of collapse. In patients transient collapse and no complications pre-hospital, they arrived in hospital with a recovered or recovering level of consciousness. There were 21 children (age <18 y) who had a collapse, who were more likely to have a seizure and cardiac arrest (Table 2).*<br>*Twenty-five patients (16%) with collapse died compared to seven (0.6%) without*

				collapse (difference: 15%: 95% CI: 8-21%; p<0.001). In 21 of the 25 patients, death was immediately associated with cardiac arrest following collapse, the remaining four did not have a cardiac arrest and died later from other complications: three from intracranial haemorrhage and one from hyperthermia. For the 42 patients with collapse and cardiac arrest, return of spontaneous circulation (ROSC) was reported in 30 patients, with the time to ROSC being significantly less in surviving patients, median 5 min (IQR: 2-6 min; range: 1-40 min; N=18), compared to those who died, median 45 min (IQR: 32-60 min; range: 15-120 min; N=12; p<0.001). The 21 patients who died following cardiac arrest were more likely to collapse in the pre-hospital setting, 13/21 (62%), rather than in hospital or with ambulance, 6/21 (29%) (Supplementary Table 2). Survivors were more likely to collapse in hospital or with ambulance (12/21; 57%).
		Case-control study—Report numbers in each exposure category, or summary measures of exposure		
		Cross-sectional study—Report numbers of outcome events or summary measures		
Main results	16	(*a*) Give unadjusted estimates and, if applicable, confounder-adjusted estimates and their precision (eg, 95% confidence interval). Make clear which confounders were adjusted for and why they were included	Table 1	
		(*b*) Report category boundaries when continuous variables were categorized	N/A	
		(*c*) If relevant, consider translating estimates of relative risk into absolute risk for a meaningful time period		
Other analyses	17	Report other analyses done—eg analyses of subgroups and interactions, and sensitivity analyses	6	As above
**Discussion**				
Key results	18	Summarise key results with reference to study objectives	7	We have shown that collapse following snakebite occurs early and is associated with more severe outcomes, including cardiac arrest and death. It occurs most commonly in brown snake envenoming and was always associated with VICC in our study. The median time to collapse was 20 minutes and it occurred within 60 minutes in 99% of cases. For this reason, 84% of collapses occurred in the pre-hospital setting and antivenom was never administered before collapse, supporting the importance of immediateearly

				resuscitation in severe snake envenoming. This was reinforced by the fact that patients who survived were more likely to receive immediate resuscitation, because they were in hospital, with ambulance officers or had immediate bystander resuscitation (Supp Table 1).
Limitations	19	Discuss limitations of the study, taking into account sources of potential bias or imprecision. Discuss both direction and magnitude of any potential bias	7	Important limitations of the study were accurate estimation of time of collapse, difficulties measuring blood pressure or other vital observations at the time of collapse and some collapses being unwitnessed. Time of collapse was based on that reported to the admitting doctor, ambulance or other health care workers. In addition, patients presenting late may have not recalled a collapse, particularly if it was not witnessed. However, the very nature of the collapse occurring early meant most patients with collapse were transported to hospital earlyimmediately. We can't comment on antivenom effectiveness for collapse, because none received it early enough, although even if it were effective, its delivery mainly in the prehospital setting seems impractical. We are not able to assess the effect of patient co-morbidities on collapse, because this information is not routinely collected for ASP. However, the majority of snakebite patients are healthy, and there was no different in age between those with and without collapse. Finally, there may be some bias in patients having collapse being more likely to be recruited to ASP.
Interpretation	20	Give a cautious overall interpretation of results considering objectives, limitations, multiplicity of analyses, results from similar studies, and other relevant evidence	7	Collapse following Australian snake envenomation occurred early, and in our study was always associated with VICC, most commonly from brown snake bites. Collapse occurs within 60 minutes post-bite and is associated with much worse outcomes, with increased rates of cardiac arrest and death, particularly if early immediate resuscitation is not instituted. Survival was associated with collapse occurring in hospital or ambulance, and with a short time to ROSC.
Generalisability	21	Discuss the generalisability (external validity) of the study results	7	
Other information				
Funding	22	Give the source of funding and the role of the funders for the present study and, if applicable, for the original study on which the present article is based	8	

*Give information separately for cases and controls in case-control studies and, if applicable, for exposed and unexposed groups in cohort and cross-sectional studies.

Note: An Explanation and Elaboration article discusses each checklist item and gives methodological background and published examples of transparent reporting. The STROBE checklist is best used in conjunction with this article (freely available on the Web sites of PLoS Medicine at http://www.plosmedicine.org/, Annals of Internal Medicine at http://www.annals.org/, and Epidemiology at http://www.epidem.com/). Information on the STROBE Initiative is available at www.strobe-statement.org.
